# Effect of glutamate infusion on NT-proBNP after coronary artery bypass grafting in high-risk patients (GLUTAMICS II): A randomized controlled trial

**DOI:** 10.1371/journal.pmed.1003997

**Published:** 2022-05-09

**Authors:** Jonas Holm, Gabriele Ferrari, Anders Holmgren, Farkas Vanky, Örjan Friberg, Mårten Vidlund, Rolf Svedjeholm

**Affiliations:** 1 Department of Thoracic and Vascular Surgery, Department of Health, Medicine and Caring Sciences, Unit of Cardiovascular Medicine, Linköping University, Linköping, Sweden; 2 Department of Cardiothoracic and Vascular Surgery, Faculty of Medicine and Health, Health Care Research Center, Örebro University, Örebro, Sweden; 3 Heart Center and Department of Public Health and Clinical Medicine, Medicine, Umeå University, Umeå, Sweden; 4 Department of Cardiothoracic Surgery, Sahlgrenska University Hospital, Gothenburg, Sweden; Oregon Health and Science University, UNITED STATES

## Abstract

**Background:**

Animal and human data suggest that glutamate can enhance recovery of myocardial metabolism and function after ischemia. N-terminal pro-brain natriuretic peptide (NT-proBNP) reflects myocardial dysfunction after coronary artery bypass surgery (CABG). We investigated whether glutamate infusion can reduce rises of NT-proBNP in moderate- to high-risk patients after CABG.

**Methods and findings:**

A prospective, randomized, double-blind study enrolled patients from November 15, 2015 to September 30, 2020, with a 30-day follow-up at 4 academic cardiac surgery centers in Sweden. Patients underwent CABG ± valve procedure and had left ventricular ejection fraction ≤0.30 or EuroSCORE II ≥3.0. Intravenous infusion of 0.125 M L-glutamic acid or saline at 1.65 mL/kg/h started 10 to 20 minutes before releasing the aortic cross-clamp, then continued for another 150 minutes. Patients, staff, and investigators were blinded to the treatment. The primary endpoint was the difference between preoperative and day-3 postoperative NT-proBNP levels. Analysis was intention to treat.

We studied 303 patients (age 74 ± 7 years; females 26%, diabetes 47%), 148 receiving glutamate group and 155 controls. There was no significant difference in the primary endpoint associated with glutamate administration (5,390 ± 5,396 ng/L versus 6,452 ± 5,215 ng/L; *p* = 0.086). One patient died ≤30 days in the glutamate group compared to 6 controls (0.7% versus 3.9%; *p* = 0.12). No adverse events linked to glutamate were observed.

A significant interaction between glutamate and diabetes was found (*p* = 0.03). Among patients without diabetes the primary endpoint (mean 4,503 ± 4,846 ng/L versus 6,824 ± 5,671 ng/L; *p* = 0.007), and the incidence of acute kidney injury (11% versus 29%; *p* = 0.005) was reduced in the glutamate group. These associations remained significant after adjusting for differences in baseline data.

The main limitations of the study are: (i) it relies on a surrogate marker for heart failure; and (ii) the proportion of patients with diabetes had almost doubled compared to the cohort used for the sample size estimation.

**Conclusions:**

Infusion of glutamate did not significantly reduce postoperative rises of NT-proBNP. Diverging results in patients with and without diabetes agree with previous observations and suggest that the concept of enhancing postischemic myocardial recovery with glutamate merits further evaluation.

**Trial registration:**

ClinicalTrials.gov https://clinicaltrials.gov/ct2/show/NCT02592824.

European Union Drug Regulating Authorities Clinical Trials Database (Eudra CT number 2011-006241-15).

## Abstract 2

Rolf Svedjeholm and colleagues investigate whether glutamate infusion can reduce rises of N-terminal pro-brain natriuretic peptide in moderate- to high-risk patients after coronary artery bypass surgery.

## Author summary

**Why was this study done?:**

- Current treatment of postoperative heart failure, the main cause for morbidity and mortality after cardiac surgery, is poorly evidence based and concerns have been raised that the marked increase in myocardial oxygen demand caused by conventional inotropic drugs may be harmful for the ischemic myocardium.
- Glutamate plays a key role for recovery of oxidative metabolism in the heart by replenishment of Krebs cycle intermediates lost during ischemia, and both animal experimental data and studies in humans suggest that glutamate can enhance recovery of myocardial metabolism and contractile function after ischemia.
- Encouraging clinical experience motivated the first GLUTAMICS trial, which was negative regarding the primary endpoint, but post hoc analyses demonstrated that the incidence of severe postoperative heart failure was significantly reduced in high-risk groups except in patients with diabetes.

**What did the researchers do and find?:**

- We studied 303 moderate- to high-risk patients undergoing surgery for ischemic heart disease (age 74 ± 7 years; females 26%, diabetes 47%) who were randomized to blinded infusion of intravenous glutamate or saline, and used the difference between preoperative and day-3 postoperative NT-proBNP levels as the primary endpoint.
- Glutamate administration was not associated with a significant reduction in the primary endpoint (5,390 ± 5,396 ng/L versus 6,452 ± 5,215 ng/L; *p* = 0.086).
- A significant interaction between glutamate and diabetes was found (*p* = 0.03) and among patients without diabetes the primary endpoint (mean 4,503 ± 4,846 ng/L versus 6,824 ± 5,671 ng/L; *p* = 0.007), and the incidence of acute kidney injury (11% versus 29%; *p* = 0.005) was reduced in the glutamate group.

**What do these findings mean?:**

- Diverging results in patients with and without diabetes agree with previous observations and suggest that the concept of enhancing postischemic myocardial recovery with glutamate merits further evaluation.

## Introduction

The contractile function of the heart is directly linked to substrate metabolism [1]. Glutamate plays a key role in myocardial metabolism during ischemia and reperfusion [2–6]. Ischemic heart disease is characterized by an increase in the myocardial consumption of glutamate [7,8]. The high fractional extraction rates of glutamate observed across the myocardium early after coronary artery bypass surgery suggest that a transient substrate deficiency occurs in these conditions [9]. In animal models, glutamate administration has been reported to facilitate the recovery of myocardial metabolism and contractile function after severe ischemia [3–6]. In cardiac surgery, glutamate has been used as an additive in cardioplegic solutions, but as cold cardioplegia has been reported to be associated with myocardial loss of glutamate other forms of administration might be preferable [10].

Intravenous infusion of glutamate during reperfusion increased myocardial uptake of glutamate and was associated with beneficial metabolic and hemodynamic effects [11,12]. Encouraging clinical experience prompted us to conduct the GLUTamate for Metabolic Intervention in Coronary Surgery (GLUTAMICS) trial [13,14]. That trial showed negative results mainly due to a high proportion of low-risk patients. Subgroup analyses of secondary endpoints suggested that glutamate could prevent or mitigate postoperative myocardial dysfunction in most risk groups, but patients with diabetes were a notable exception [14].

N-terminal pro-brain natriuretic peptide (NT-proBNP) is an established biomarker of heart failure in cardiology practice [15]. NT-proBNP has also been found to reflect heart failure after cardiac surgery [16–21]. Postoperative heart failure remains the main cause for postoperative morbidity and mortality [22–24]. Interventions that can reduce the need for inotropes are desirable as the increased myocardial oxygen demand associated with these drugs may harm the ischemic heart [25,26]. Therefore, we decided to investigate whether intravenous glutamate infusion can mitigate postoperative myocardial dysfunction in moderate- to high-risk patients undergoing coronary artery bypass surgery (CABG) [22–24]. We hypothesized that glutamate would enhance recovery of the postischemic heart and, consequently, glutamate administration would reduce the postoperative rise in NT-proBNP.

## Materials and methods

### Study design

The GLUTAmate for Metabolic Intervention in Coronary Surgery II (GLUTAMICS II) trial was investigator initiated and designed as a prospective, externally randomized, placebo-controlled, double-blind trial with parallel assignment to glutamate or placebo (saline). The study and its amendments were approved by the Swedish Medical Products Agency (Dnr 151:2011/96689; Dnr 5.1-2015-77379; Dnr 5.1-2016-62288; Dnr 5.1-2017-71108). The latest study protocol is presented in the S1 Protocol. The trial was registered at the European Union Drug Regulating Authorities Clinical Trials Database (Eudra CT number 2011-006241-15) and at ClinicalTrials.gov. https://clinicaltrials.gov/ct2/show/NCT02592824.

This trial is reported according to the Consolidated Standards of Reporting Trials statement (S1 Checklist).

### Ethics

The study and its amendments were approved by the Regional Ethical Review Board in Linköping: Dnr 2011/498-31 (January 25, 2012); Dnr 2015/333-32 (October 7, 2015); Dnr 2016/329-32 (September 1, 2016). All patients were enrolled in the study after providing written informed consent. The study was performed according to the Helsinki Declaration of Human Rights.

### Study population

Screening was done by the clinical investigators at 4 academic cardiac surgery centers in Sweden. Two other centers agreed to participate but did not contribute. We included 303 patients in the final analysis out of 314 initially randomized patients between November 15, 2015 and September 30, 2020, with a 30-day follow-up. No crossovers occurred but 8 patients were excluded because of intraoperative exclusion criteria and in 3 instances case record forms were lost. Analysis was done according to intention to treat. A CONSORT diagram is given in Fig A in S1 Supporting information.

Patients were eligible for the study if they were accepted for on-pump CABG, due to least 2-vessel disease or left main stenosis, with or without a concomitant procedure. Moreover, patients had to be at moderate to high risk of postoperative heart failure. This implied either a preoperative left ventricular ejection fraction ≤0.30 or a European System for Cardiac Risk Evaluation II (EuroSCORE II) score ≥3.0, with at least one of the following cardiac or procedure-related risk factors: an urgent or emergency procedure, Canadian Cardiovascular Society (CCS) class IV angina, left ventricular ejection fraction ≤0.50, myocardial infarction within 90 days prior, or a concomitant aortic or mitral valve procedure.

Exclusion criteria were age >85 years, ambiguous food allergy known to have triggered shortness of breath, headache or flushing, previous cardiac surgery, informed consent not possible due to a critical condition or other reason, preoperative use of mechanical circulatory assistance, renal failure with preoperative dialysis or calculated creatinine clearance <30 mL/min, surgery without heart–lung machine, concomitant maze procedure, surgery on the ascending aorta, and surgery on both the aortic and mitral valves.

Patients were monitored by a professional external monitoring team. The team confirmed the correct inclusion, provision of informed written consent, primary and secondary endpoints, and records of adverse events. Due to Coronavirus Disease 2019 (COVID-19) and travel restrictions, 9 patients operated in Gothenburg could not be monitored, but the data were double checked by a research nurse.

### Interventions

Patients were randomly allocated in a 1:1 ratio to a blinded intravenous infusion of either a 0.125 M L-glutamic acid solution or saline, at a rate of 1.65 mL/kg body weight/h. The infusion was started 10 to 20 minutes before the anticipated release of the aortic cross-clamp. After declamping, the infusion was continued for another 2 hours, then the infusion rate was halved, and an additional 50 mL was infused. The maximum volume infused to any patient was 500 mL of study solution.

### Glutamate solution

A total of 500 mL 0.125 M solution of L-glutamic acid with pH 6.0 and 280 mosmol/kg containing L-glutamic acid 9.2 g, NaCl 0.8 g, H_2_O ad 500 mL, and NaOH quantum satis.

The production of glutamate solution and quality control was done by Apoteket AB, Produktion & Laboratorier (APL), Box 6124, SE 90621 Umeå, Sweden (www.apl.se).

### Outcomes and safety endpoints

The primary outcome was the postoperative increase in circulating NT-proBNP from preoperative levels to the third postoperative day.

Secondary outcomes were the absolute plasma levels of NT-proBNP on the first and third postoperative day.

The safety endpoints were postoperative stroke within 24 hours, postoperative mortality within 30 days, and suspected unexpected adverse reactions (SUSARs) within 24 hours.

### NT-proBNP analyses

Venous blood samples for NT-proBNP were drawn at 3 time points: preoperatively, the first and third morning after surgery. Plasma NT-proBNP was measured on a Roche Cobas platform (Roche Diagnostics, Basel, Switzerland). The assay had an effective measuring range of 5 to 35,000 ng/L. Samples were diluted and reanalysed for NT-proBNP values exceeding 35,000 ng/L. The inter-assay coefficient of variation was <5%. Assays were done at the laboratories of the 4 participating university hospitals in Linköping, Örebro, Umeå, and Gothenburg. These laboratories are accredited by SWEDAC (www.swedac.se), and the precision of analytical methods are validated and verified against each other through the external national analysis quality system Equalis (www.equalis.se).

### Sample size

Sample size was estimated by an external professional statistician and based on available data from the first GLUTAMICS trial [14,27]. The analysis suggested that 141 patients should be included in each group (80% power; 5% risk level; 2-sided *t* test assuming unequal variances), but to account for missed samples, the aim was set to a total of 310 patients. Interim analysis of the first 160 patients used an adaptive design, addressing postoperative increase of NT-proBNP from preoperative state to the first postoperative day. Interim analysis supported the initial calculation and addressed both futility and modification of sample, which according to initial sample size calculation was assumed to be 2/3 of the sample suggested by the surrogate endpoint. The number of missed samples was lower than anticipated, and patient inclusion was terminated on September 30, 2020.

### Randomization and blinding

Underlying heart disease and cardiac procedure can influence the levels of natriuretic peptides [28]. Randomization was, therefore, stratified for isolated CABG and CABG with additional valve procedure. The randomization was computer generated by the producer of the study solutions (APL, Sweden) in variable block sizes. Each infusion bottle contained 2 labels with the randomization number, one was a self-adhesive label that could be removed and transferred to the case report form (CRF) to ensure that each study participant was matched to the correct information. The label was transferred to the CRF in the operating room, just before the intravenous solution was connected, at the point when the patient was randomized and included in the trial. Patients, staff, and investigators were blinded to the infused treatment (clear transparent solutions). Allocation was concealed until the study was terminated by keeping the randomization codes at APL, Sweden. For safety reasons, the sponsor had access to sealed opaque envelopes to permit intervention to be revealed in cases of SUSAR, mortality, or stroke within 24 hours of surgery. The external monitoring team checked all envelopes at the end of the study. After the study had been terminated and the data base had been locked, APL provided the randomization codes to the statistician who analyzed endpoints and key study data before the investigators were given access.

### Clinical management

Clinical management was standardized and similar at the participating centers with minor differences. Cardiopulmonary bypass (CPB) and aortic cross-clamping were employed. Cold blood cardioplegia was used for myocardial protection. Inotropes were given at the discretion of the attending physician. Intraoperative and postoperative glycemic control was employed at all participating centers with a p-glucose target of 5 to 10 mmol/L. Insulin infusion was started at p-glucose 8 mmol/L (Örebro) or 10 mmol/L (other centers). Further details are given in the Clinical management in S1 Supporting information.

### Definitions

Diabetes was defined as patients with a diagnosis of diabetes mellitus on admission to the cardiac surgical department.

Severe left ventricular dysfunction corresponds to an ejection fraction of ≤0.30.

Postoperative mortality was defined as mortality within 30 days of surgery.

Postoperative stroke was defined as neurological or cognitive deficit with a cerebral injury verified on computed tomography (CT) scan. All suspected cases of stroke underwent CT scan.

Stroke ≤24 hours of surgery was defined as a stroke that occurred within 24 hours of surgery or signs of a stroke, when first assessable in deeply sedated patients on a ventilator.

Acute kidney injury was defined according to RIFLE criteria as a postoperative increase in plasma creatinine by at least 50%.

Postoperative myocardial injury was measured with creatine kinase-MB isoenzyme (CK-MB) on the first postoperative morning. Different troponin analyses were used at the participating sites and, hence, are not presented.

### Statistical analysis

Statistical analyses of endpoints were performed by an external statistician before randomization codes became available to the investigators. The initial statistical analysis plan is presented in the Supplementary Methods in S1 Supporting information and the S1 Protocol. Two-sided Student *t* test or Mann–Whitney U test were used for between group comparisons of continuous variables. Levene’s test was used to assess equality of variances. Categorical variables were analyzed with Fisher’s exact test. Depending on the use of test, results are presented as means ± standard deviation, medians and interquartile range [IQR], or numbers and percentages (%). The mean difference between groups for the primary endpoint is given with 95% confidence interval. The primary endpoint was also analyzed after logarithmic transformation.

Exploratory analyses were done with ANOVA, multiple linear regression, ANCOVA, and logistic regression. Statistical significance was defined as *p* < 0.05. Statistical analyses were performed with computerized statistical packages (Minitab 19, Minitab Statistical Software, LCC, State College, Pennsylvania, United States of America and IBM SPSS version 27, IBM, Armonk, New York, USA).

## Results

### Patient characteristics

We analyzed a total of 303 patients, 148 in the glutamate group and 155 in the control group. The mean ages were, respectively, 73 ± 7 years versus 75 ± 7 years; *p* = 0.004. The glutamate group had a lower mean EuroSCORE II than the control group (4.6 ± 2.1% versus 5.2 ± 2.5%; *p* = 0.02), and a higher estimated creatinine clearance (69 ± 24 mL/min versus 63 ± 23 mL/min; *p* = 0.01). The proportions of patients with diabetes were high in both groups (49% versus 46%). Detailed patient characteristics are given in Table 1.

**Table 1:** Preoperative characteristics of the glutamate group and the control group (saline).

Variables	Glutamate (*n* = 148)	Control (*n* = 155)
Age, years	73 ± 7	75 ± 7
Female sex, no. (%)	36 (24)	43 (28)
BMI, kg•m^2^	28 ± 5	28 ± 4
EuroSCORE II, %	4.6 ± 2.1	5.2 ± 2.5
Diabetes, no. (%)	72 (49)	71 (46)
Hypertension, no. (%)	113 (76)	119 (77)
COPD, no. (%)	23 (16)	21 (14)
Peripheral arterial disease, no. (%)	26 (18)	35 (23)
Cerebrovascular disease, no. (%)	10 (7)	16 (10)
p-Creatinine, μmol•L^‒1^	99 ± 26	105 ± 30
eCrCl, mL•min^‒1^	69 ± 24	63 ± 23
NT-proBNP, ng•L^‒1^	2,680 ± 4,595	2,354 ± 3,124
Left main stenosis, no. (%)	56 (38)	62 (40)
AMI ≤3 weeks, no. (%)	70 (47)	79 (51)
CCS IV, no. (%)	22 (15)	30/154 (19)
Atrial fibrillation, no. (%)	23 (16)	23 (15)
Severe LV dysfunction, no. (%)	45 (30)	40/154 (26)

AMI ≤3 weeks, acute myocardial infarction within 3 weeks of surgery; BMI, body mass index; CCS, Canadian Cardiovascular Society; COPD, chronic obstructive pulmonary disease; eCrCl, estimated creatinine clearance according to Cockcroft–Gault formula; EuroSCORE II, European System for Cardiac Operative Risk Evaluation II; LV, left ventricular; STEMI, ST-elevation myocardial infarction.

### Primary outcome

A nonsignificant reduction of the primary endpoint was seen in the glutamate group compared to the control group (5,390 ± 5,396 ng/L versus 6,452 ± 5,215 ng/L; *p* = 0.086) (Table 2).

**Table 2:** Intraoperative and postoperative characteristics of the glutamate group and the control group (saline).

Variables	Glutamate (*n* = 148)	Control (*n* = 155)	*P-*value
Urgent/emergent procedure, no. (%)	98 (66)	99 (64)	0.72
Number of bypasses	3.6 ± 1.1	3.5 ±1.0	0.23
Additional valve procedure, no. (%)	32 (22)	33 (21)	1
Aortic cross-clamp time, minute	71 ± 30	69 ± 33	0.68
CPB time, minute	109 ± 43	110 ± 40	0.84
NT-proBNP POD1, ng•L^‒1^	4,438 ± 4,879	4,420 ± 4,236 *n* = 153	0.97
NT-proBNP POD3, ng•L^‒1^	8,055 ± 7,546 *n* = 145	8,804 ± 6,606 *n* = 150	0.36
NT-proBNP POD3-Pre, ng•L^‒1^	5,390 ± 5,396 *n* = 145	6,452 ± 5,215 *n* = 150	0.09
Log NT-proBNPOD3-Pre*	7.80 ± 3.13	8.29 ± 1.99	0.11
CK-MB POD1, μg•L^‒1^	16 [12–29] *n* = 128	18 [13–30] *n* = 134	0.75
ICU stay, days	1 [1–2]	1 [1–2]	0.47
Ventilation time, hour	4.0 [2.5–6.9]	4.3 [2.8–7.2]	0.41
Ventilation time >48 hours, no. (%)	10 (6.8)	7 (4.5)	0.46
IABP, no. (%)	2 (1.3)	2 (1.3)	1
Reoperation bleeding, no. (%)	11 (7.4)	17 (11)	0.33
Postoperative AFib, no. (%)	62 (42)	70 (45)	0.64
Postop stroke ≤ 24 hours, no. (%)	0 (0)	4 (2.6)	0.12
AKI, no. (%)	26 (18)	40 (27)	0.10
SUSARs, no. (%)	0 (0)	0 (0)	1
Mortality ≤30 days, no. (%)	1 (0.7)	6 (3.9)	0.12

AFib, atrial fibrillation; AKI, acute kidney injury; CK-MB, creatine kinase-MB isoenzyme; CPB, cardiopulmonary bypass; IABP, intra-aortic balloon pump; ICU, intensive care unit; POD, postoperative day; SUSARs, suspected unexpected serious adverse reactions.

*Logarithmic transformation of the difference from preoperative NT-proBNP to postoperative day 3. Some patients decreased in NT-proBNP, and for the negative values, the absolute log was taken and then multiplied with ‒1.

### Secondary outcomes

Postoperative NT-proBNP levels on the first and third postoperative days did not differ significantly between the groups (Table 2).

### Safety outcomes

One patient died ≤30 days of surgery in the glutamate group compared to 6 in the control group (0.7% versus 3.9%; *p* = 0.12). The incidence of stroke ≤24 hours did not differ significantly between groups (0% versus 2.6%; *p* = 0.12). No SUSAR was reported (Table 2).

### Exploratory outcomes

A doubling of the proportion of patients with diabetes compared to the cohort used for sample size estimation and a significant interaction between diabetes and glutamate affecting the primary endpoint (Fig B in S1 Supporting information; *p* = 0.03) and warranted post hoc analyses (Tables 3 and 4).

**Table 3:** Preoperative characteristics of patients without diabetes in the glutamate group and in the control group (saline).

Variables	Glutamate (*n* = 76)	Control (*n* = 84)
Age, years	74 ± 7	76 ± 6
Female sex, no. (%)	19 (25)	23 (27)
BMI, kg•m^2^	27 ± 4	27 ± 4
EuroSCORE II, mean (SD), %	4.3 ± 1.6	4.8 ± 2.4
Diabetes, no. (%)	0 (0)	0 (0)
Hypertension, no. (%)	51 (67)	59 (70)
COPD, no. (%)	13 (17)	12 (14)
Peripheral arterial disease, no. (%)	13 (17)	19 (23)
Cerebrovascular disease, no. (%)	5 (7)	9 (11)
p-Creatinine, μmol•L^‒1^	96 ± 25	103 ± 27
eCrCl, mL•min^‒1^	68 ± 23	62 ± 21
NT-proBNP, ng•L^‒1^	2,405 ± 3,559	2,065 ± 1,902
Left main stenosis, no. (%)	33 (43)	31 (37)
AMI ≤3 weeks, no. (%)	29 (38)	41 (49)
CCS IV, no. (%)	12 (16)	15 (18)
Atrial fibrillation, no. (%)	13 (17)	13 (16)
Severe LV dysfunction, no. (%)	26 (34)	24/83 (29)

AMI ≤3 weeks, acute myocardial infarction within 3 weeks of surgery; BMI, body mass index; CCS, Canadian Cardiovascular Society; COPD, chronic obstructive pulmonary disease; eCrCl, estimated creatinine clearance according to Cockcroft–Gault formula; EuroSCORE II, European System for Cardiac Operative Risk Evaluation II; LV, left ventricular; STEMI, ST-elevation myocardial infarction.

**Table 4:** Intraoperative and postoperative characteristics of patients without diabetes in the glutamate group and in the control group (saline).

Variables	Glutamate (*n* = 76)	Control (*n* = 84)	*P-*value
Urgent/emergent procedure, no. (%)	40 (53)	47 (56)	0.75
Number of bypasses	3.6 ± 1.0	3.4 ± 0.9	0.21
Additional valve procedure, no. (%)	23 (30)	21 (25)	0.48
Aortic cross-clamp time, minute	72 ± 29	71 ± 35	0.93
CPB time, minute	111 ± 41	112 ± 46	0.84
NT-proBNP POD1, ng•L^‒1^	3,994 ± 3,607	4,340 ± 3,417	0.53
NT-proBNP POD3, ng•L^‒1^	6,884 ± 4,279 *n* = 75	8,911 ± 6,605 *n* = 83	0.03
NT-proBNP POD3-Pre, ng•L^‒1^	4,503 ± 4,846 *n* = 75	6,825 ± 5,671 *n* = 83	0.007
Log NT-proBNPOD3-Pre*	7.22 ± 4.22	8.52 ± 0.53	0.01
CK-MB POD1, μg•L^‒1^	16 [11–34]	19 [13–33]	0.75
ICU stay, days	1 [1–3]	1 [1–2]	0.40
Ventilation time, hour	3.5 [2.5–5.1]	4.2 [2.5–7.2]	0.31
Ventilation time >48 hours, no. (%)	4 (5.3)	5 (6.0)	1
IABP, no. (%)	1 (1.3)	1 (1.2)	1
Reoperation bleeding, no. (%)	5 (6.6)	9 (11)	0.41
Postoperative AFib, no. (%)	35 (46)	37 (44)	0.87
Postop stroke ≤24 hours, no. (%)	0 (0)	2 (2.4)	0.50
AKI, no. (%)	8 (11)	24 (29)	0.005
Mortality ≤30 days, no. (%)	0 (0)	1 (1.2)	1

AFib, atrial fibrillation; AKI, acute kidney injury; CK-MB, creatine kinase-MB isoenzyme; CPB, cardiopulmonary bypass; IABP, intra-aortic balloon pump; ICU, intensive care unit; POD, postoperative day.

*Logarithmic transformation of the difference from preoperative NT-proBNP to postoperative day 3. Some patients decreased in NT-proBNP, and for the negative values, the absolute log was taken and then multiplied with ‒1.

No significant interactions with regard to the primary endpoint were found between glutamate and other baseline variables in the study (including reported subgroups).

Among patients without diabetes, a significant reduction in the increase of NT-proBNP from preoperative levels to the third postoperative day was found (mean 4,503 ± 4,846 ng/L versus 6,824 ± 5,671 ng/L; *p* = 0.007). The mean difference between glutamate group minus control group was ‒2,321 (95% confidence interval ‒3,988 to ‒655) ng/L.

The incidence of acute kidney injury was also significantly lower in the glutamate group compared to controls (11% versus 29%; *p* = 0.005).

Demographics and periprocedural risk factors did not differ significantly between treatment groups among patients without diabetes. We adjusted for age, estimated creatinine clearance, and EuroSCORE II in multivariable analyses. Glutamate was found to be significantly associated with a reduction in the postoperative rise in NT-proBNP and a lower risk of acute kidney injury (Tables A and B in S1 Supporting information).

Addressing variables significantly associated with the primary endpoint among patients without diabetes, we found that glutamate remained in the final model together with preoperative NT-proBNP, renal function, and additional valve procedure (Table C in S1 Supporting information).

The results after logarithmic transformation of the primary endpoint are given in Tables 2 and 4. A sensitivity analysis addressing site and additional valve procedure with logarithmic transformation of the difference between preoperative NT-proBNP and NT-proBNP postoperative day 3 is presented in Table D in S1 Supporting information.

Preoperative, intraoperative, and postoperative data for patients with diabetes are given in Tables E and F in S1 Supporting information. No effect of glutamate on the primary endpoint was seen among patients with diabetes.

Preoperative, intraoperative, and postoperative data for patients undergoing additional valve procedure are given in Tables G and H in S1 Supporting information.

## Discussion

The main finding of this study in moderate- to high-risk patients undergoing CABG is that intravenous glutamate infusion during the first hours of reperfusion did not significantly reduce rise of plasma NT-proBNP from preoperative level to the third postoperative day. However, a significant interaction was found between diabetes and glutamate that affected the primary endpoint.

Among patients without diabetes, infusion of glutamate was associated with a significant reduction in the primary endpoint compared to controls. The multivariable analyses, which adjusted for confounders and baseline imbalances, seem to support a role for glutamate after CABG among patients without diabetes. These results agree with the first GLUTAMICS trial, which showed that intravenous glutamate reduced the risk of developing severe postoperative heart failure by approximately half in all studied risk groups undergoing CABG, except in patients with diabetes [14]. Half-way into the first GLUTAMICS trial, a feasibility study of NT-proBNP as a surrogate marker for postoperative heart failure was added and the results partially agree with the current trial [27,29]. Post hoc analyses demonstrated a significant reduction in the postoperative rise of NT-proBNP in a glutamate-treated high-risk cohort, which included patients with diabetes [27]. When the current study was designed to confirm these findings in risk patients, it was not anticipated that the proportion of patients with diabetes would almost double to 47% compared with the cohort used for sample size estimation [14,27].

A significant reduction in the incidence of acute kidney injury was also found in the glutamate group among patients without diabetes. Renal function is a sensitive clinical marker of postoperative heart failure, and acute kidney injuries are not uncommon after cardiac surgery [30–32]. Although postoperative renal dysfunction is typically transient, there is evidence that even mild to moderate cases may be associated with an impaired long-term prognosis [31,32]. Available data on other clinical variables such as use of inotropes, echocardiographic findings, and readmissions were not sufficiently detailed for meaningful analyses.

No adverse effects related directly to glutamate were observed. Regarding safety endpoints, we observed only 1 death within 30 days (0.7%) and no stroke within 24 hours in the glutamate group.

In this study, the purpose of glutamate infusion was to enhance the natural postischemic recovery of cardiac oxidative metabolism and contractile function. Regardless of supplementation, the heart is utilizing increased amounts of glutamate in association with ischemia [7,8]. Early after CABG, a strong correlation between arterial levels of glutamate and myocardial uptake has been observed [9]. Available data from patients undergoing CABG suggest that some degree of myocardial metabolic derangement is required for glutamate supplementation to make a difference [11,12,33].

The dosage of glutamate was based on a study after CABG looking at correlations between infusion rate, arterial levels, and myocardial uptake of glutamate [34]. The duration of treatment was based on clinical experience regarding hemodynamic recovery in high-risk patients [13].

According to studies in a rat heart model and biopsies from the human heart glutamate, administration does not benefit diabetic hearts, due to down-regulation of mitochondrial glutamate transporter EAAT1 resulting in increased intracellular glutamate levels and a reduced capacity for glutamate supported respiration [35,36]. Those findings agree with the divergent results in patients with and without diabetes in the first and second GLUTAMICS trials [14].

This study has limitations, other than the inclusion of a high proportion of patients with diabetes. It might have been preferable to use clinical hard endpoints, such as mortality or clinical heart failure postoperatively, rather than a biomarker. However, those endpoints would have required a much larger sample size. Given the lack of generally accepted criteria for postoperative heart failure, a biomarker could be preferable for unbiased assessment of myocardial dysfunction after cardiac surgery [29]. The rise in postoperative NT-proBNP has been shown to be associated with postoperative heart failure, morbidity, and mortality [16,18–21,37]. We found a good agreement between the hemodynamic criteria used for postoperative heart failure in the first GLUTAMICS trial and postoperative NT-proBNP levels [29]. A larger sample size could have prevented imbalances observed for some risk factors, but multivariable analyses suggest that they did not explain the results observed among patients without diabetes.

The results of this trial are at best hypothesis generating but suggest that future studies on the effect of glutamate on postischemic myocardial recovery in humans should focus on patients without diabetes. If the findings of this and the first GLUTAMICS trial can be confirmed, the clinical implications could be profound reducing mortality and morbidity after surgery for ischemic heart disease. To address generalizability, studies on critical myocardial ischemia in settings other than cardiac surgery might be worthwhile.

To summarize, the GLUTAMICS II trial fell short of showing a significant effect of intravenous glutamate on postoperative rises in NT-proBNP levels in moderate- to high-risk patients that underwent CABG. Diverging results in patients with and without diabetes agree with previous observations and suggest that the concept of enhancing postischemic myocardial recovery with glutamate merits further evaluation.

## Supporting information

S1 Checklist CONSORT Checklist. (DOCX)

S1 Protocol Study protocol. (DOCX)

S1 Supporting information Supporting tables and figures. (DOCX)

## Abbreviations

AKI: acute kidney injury
CABG: coronary artery bypass grafting
CCS: Canadian Cardiovascular Society
CK-MB: creatine kinase-MB isoenzyme
COVID-19: Coronavirus Disease 2019
CPB: cardiopulmonary bypass
CRF: case report form
CT: computed tomography
EuroSCORE II: European System for Cardiac Operative Risk Evaluation II
GLUTAMICS: GLUTAmate for Metabolic Intervention in Coronary Surgery
IABP: intra-aortic balloon pump
ICU: intensive care unit
IQR: interquartile range
NT-proBNP: N-terminal pro-brain natriuretic peptide
SUSAR: suspected unexpected adverse reaction
