# Supplementary material for: Effect of glutamate infusion on NT-proBNP after coronary artery bypass grafting in high-risk patients (GLUTAMICS II): A randomized controlled trial
Source: PLoS Med. 2022 May 9;19(5):e1003997. doi: 10.1371/journal.pmed.1003997 (PMC9126383; doi:10.1371/journal.pmed.1003997)
Supplement: S1 Protocol — (DOCX) [file pmed.1003997.s002.docx]

Metabolic intervention with glutamate in surgery for ischemic heart disease:(GLUTA*MICS* II = GLUTAmate for Metabolic Intervention in Coronary Surgery II)

**EudraCT number:** 2011-006241-15

**Sponsor's Protocol Code Number** GLUTAMICS2

**Sponsor and Principal investigator:** Rolf Svedjeholm (consultant cardiac surgeon, professor) Dept of Thoracic and Vascular Surgery, Linköping University Hospital, SE 581 85 Linköping, Sweden

Telephone: +46101034825

E-mail: rolf.svedjeholm@regionostergotland.se

**Participating sites:**

Thorax-kärlkliniken, Universitetssjukhuset, Linköping

Thoraxkliniken, Universitetssjukhuset, Örebro

Thoraxkliniken, Sahlgrenska Universitetssjukhuset, Göteborg.

Thoraxkirurgiska enheten, Norrlands Universitetssjukhus, Umeå

Verksamhetsområdet Thoraxkirurgi och Anestesi, Akademiska sjukhuset, Uppsala*

Thoraxcentrum, Blekingesjukhuset, Karlskrona*

*No contribution

**Synopsis**

GLUTAMICS II (EudraCT number: 2011-006241-15) is an externally randomized, placebo-controlled study with parallel study groups and double-blind masking. The study drug is a 0.125M solution of glutamate given intravenously in association with coronary artery bypass surgery. Placebo consists of a normal saline.

The aim is to confirm findings from subgroups in a previous study GLUTAMICS and demonstrate that glutamate infusion reduces the risk of postoperative heart failure.

The primary endpoint is the increase of NT-proBNP, a biomarker for heart failure, from preoperative level the day before surgery to the third postoperative day. Secondary endpoints are the absolute levels of NT-proBNP on the first and third postoperative day.

The study population consists of patients with at least moderate preoperative risk due to cardiac risk factors undergoing coronary artery bypass surgery (CABG) of at least two-vessel disease with or without concomitant procedure. Specifically, moderate preoperative risk due to cardiac risk factors implies at least one prespecified cardiac risk factor contributing to EuroSCORE II ≥ 3.0 or severe left ventricular dysfunction (Left ventricular ejection fraction ≤ 0.30) regardless of EuroSCORE II.

Randomization will be stratified for patients undergoing isolated CABG and those undergoing CABG with a concomitant procedure.

Exclusion criteria: patients with ambiguous food allergy known to have triggered shortness of breath, headache or flushing, age > 85 years, previous cardiac surgery, informed consent not possible because of critical condition, linguistic or other reasons, preoperative use of mechanical circulatory assist, preoperative dialysis or calculated GFR < 30 mL/min, surgery without heart-lung machine (off-pump), concomitant Maze-procedure, surgery of ascending aorta, surgery of both aortic and mitral valve.

310 patients are planned to be included and interim analysis will be conducted after 160 patients. The study is anticipated to start in the middle of October 2015 and to be completed within 2 years.

**Background**

Postoperative heart failure is the leading cause of extended intensive care, multiple organ failure and death after cardiac surgery(1). Conventional treatment of postoperative heart failure with inotropic drugs improves hemodynamics but at the prize of excessive increase in myocardial oxygen demand, which could aggravate the underlying myocardial injury(2).

Myocardial metabolism in patients with coronary artery disease is characterized by an increased uptake of the amino acid glutamate(3, 4). This amino acid plays a key role in the metabolism of cardiomyocytes. In animal experiments glutamate improves myocardial tolerance to ischemia*(5, 6). After ischemia glutamate plays an important role for replenishment of Krebs cycle metabolites that have been depleted in the mitochondria and can thereby enhance normalization of the oxidative metabolism, which is disturbed after severe ischemia(7-9).

* *The suggested biochemical explanation is that glutamate can contribute to anaerobic energy production in the Krebs cycle through metabolism of α-ketoglutarate to succinate, and that anaerobic glycolysis is enhanced by the malate-aspartate shuttle by maintenance of the redox potential in the cytosol. Accumulation of lactate is inhibited by transamination of pyruvate to alanine.*

A positive effect of glutamate on the metabolic and functional recovery of the heart has also been found in small studies in humans(10, 11). In light of this and encouraging clinical experience, which compare favorably with the literature both regarding survival and heart failure related complications, the first GLUTAMICS trial was initiated(12).

In this multi-center study 861 patients undergoing CABG for acute coronary syndrome were randomized to intravenous infusion of glutamate or placebo (normal saline). The primary endpoint was a composite of postoperative mortality (≤ 30 days), perioperative myocardial infarction and left ventricular failure on weaning from the heart-lung machine. The results did not show any difference regarding the primary endpoint between the groups(13).

Regarding secondary endpoints significant differences were observed suggesting a beneficial effect on myocardial recovery after ischemia. Significantly fewer patients developed severe postoperative heart failure that prolonged intensive care or required treatment with intra-aortic balloon pump. The presumed effect of glutamate infusion was observed in the patients with CCS class IV angina, which constituted a prespecified subgroup. A relative risk reduction exceeding 50% was also observed in other high-risk groups (table 1) with the exception of diabetics.

A potential advantage with glutamate is that it works by enhancing normal recovery mechanisms, which should be less stressful for the heart than conventional treatment with inotropic drugs, which increase myocardial oxygen demand and thereby can aggravate underlying myocardial injury(2). Accordingly, we observed that the patients who fulfilled criteria for heart failure at weaning from the heart-lung machine had a significantly shorter ICU stay and a lower incidence of renal failure if they had received glutamate (table 2).

The first GLUTAMICS trial was originally planned for patients with unstable angina CCS class IV but the inclusion criteria were widened to include all patients with acute coronary syndrome, which resulted in inclusion of many low-risk patients which diluted the results and reduced the power of the study. We also observed that the primary endpoint would have benefited from a more robust design with regard to the criteria for heart failure due to liberal use of preemptive inotropes.

Another issue the first GLUTAMICS trial had to address was the lack of universally accepted criteria for the diagnosis of postoperative heart failure. This was done by using a blinded endpoints committee relying on prespecified criteria. Halfway into the trial NT-proBNP became available and a substudy was initiated to blindly evaluate this biomarker for heart failure against the heart failure classifications made by the endpoints committee. Our analyses demonstrated that postoperative high NT-proBNP levels were associated with postoperative heart failure (Figure 1). This implies that postoperative NT-proBNP could be used to evaluate prevention and treatment of postoperative heart failure after cardiac surgery.

The results suggest that intravenous glutamate potentially provides an important and gentle way to enhance myocardial recovery after ischemia, which also might prove useful in cardiology practice. However, the results from the first GLUTAMICS trial have received little attention due to the fact that the positive results were secondary endpoints in subgroups. For intravenous glutamate infusion to receive acceptance as treatment and prevention of heart failure after CABG the results need to be confirmed in future studies.

Our initial aim with this study was to conclusively demonstrate in a larger cohort (n=1400) that intravenous glutamate reduces the risk of severe heart failure after CABG in patients with CCS class IV angina or moderate to severe reduction of left ventricular ejection fraction (LVEF ≤ 0.45). Due to funding the GLUTAMICS II study has been modified and downscaled to rely on NT-proBNP as a marker of heart failure. The advantage with this redesign is that fewer patients are required and that the primary endpoint can be reproduced in future studies. Inclusion criteria have been slightly widened but the study focuses on risk patients in need of CABG. Patients accepted for CABG of at least two vessel disease with or without concomitant procedure and deemed preoperatively to carry at least a moderately increased risk for postoperative mortality (EuroSCORE II ≥ 3.0) will be considered for inclusion. Sample size estimations have been performed by an external professional statistician based on data from patients with EuroSCORE II ≥ 3.0 in the first Glutamics-trial.

**Aims**

The aim of this clinical trial is to confirm that intravenous glutamate infusion reduces the risk of postoperative heart failure in patients undergoing CABG by demonstrating mitigated increase of NT-proBNP, a biomarker for heart failure, postoperatively.

**Trial design**

Externally randomized, placebo-controlled trial with parallel study groups and double-blind masking. Normal saline, which like the glutamate solution is a clear solution, will be used as placebo.

**Study population**

- Patients accepted for CABG of at least two vessel disease or left main stenosis with or without a concomitant procedure and who either have:
  - EuroSCORE II ≥ 3.0 to which at least one of following risk factors contribute
    - LVEF ≤ 0.50
    - CCS-class IV
    - Myocardial infarct ≤ 90 days
    - Emergent or urgent procedure
    - CABG + aortic or mitral valve procedure
  - Left Ventricular Ejection Fraction ≤ 0.30 regardless of EuroSCORE II
- The procedure has to be done with heart-lung machine (on-pump)

These patients will be informed about the trial and asked to participate. Patients who accept participation and are operated with the aid of heart-lung machine will be included in the study.

Exclusion criteria: patients with ambiguous food allergy known to have triggered shortness of breath, rash or flushing, age >85 years, previous cardiac surgery, informed consent not possible because of critical condition, linguistic or other reason, renal failure with preoperative dialysis or calculated GFR < 30 mL/min, preoperative use of mechanical circulatory assist, surgery without heart-lung machine (off-pump), concomitant Maze-procedure for atrial fibrillation, surgery of ascending aorta, surgery of both aortic and mitral valve.

The participating centers Linköping, Örebro, Umeå, Gothenburg, Uppsala and Karlskrona are the only cardiac surgical centers within their region and thus account for all cardiac surgical procedures. To evaluate sampling bias the aim is to use the databases from each center.

**Research plan**

Randomized double-blind study on 310 patients fulfilling inclusion criteria. Randomization will be stratified for those undergoing isolated CABG and those undergoing CABG with concomitant procedure.

Half of the patients will receive glutamate infusion intravenously (1.65 mL/ kg bodyweight and hour of a 0.125M L-glutamic acid solution), which will be started 10-20 minutes before anticipated release of aortic cross-clamp and the start of reperfusion. The infusion will be given for 2 hours after release of aortic cross-clamp. Thereafter another 50 mL will be given with the infusion rate halved. The maximum total given will not exceed 500 mL. If the volume is insufficient due to high body weight or extension of cross-clamp time the last 50 mL should be given at half the original infusion rate. The remaining half of the patients will receive normal saline in a similar way.

**Specification of outcomes**

Primary endpoint:

The primary endpoint is postoperative increase of NT-proBNP from the day before surgery to the third postoperative day.

.

*Secondary endpoints and substudies*

Secondary endpoints are the absolute levels of NT-proBNP on postoperative day 1 and day 3.

**Subgroup analyses and substudies**

Patients undergoing isolated CABG or CABG with concomitant procedure will also be analyzed separately.

Blood samples from the three sampling time points are planned to be frozen to permit analysis of novel markers of heart failure and myocardial injury in the future.

Minor substudies may arise but will be specified and reported to the Swedish MPA and ClinicalTrials.gov before they are started.

*Safety variables*

Postoperative mortality (30 days + in-hospital), stroke within 24 hours of surgery and SUSAR.

**Sample size estimation and statistics**

The sample size is based on available results on NT-proBNP from the first GLUTAMICS-trial.

In that study the following increase of NT-proBNP was observed in patients undergoing CABG or CABG with concomitant procedure who fulfilled inclusion criteria for the planned trial from preoperative values to postoperative day 3 (mean ± Standard Deviation)

Glutamate (n=71): 5261 ± 4409

Placebo (n=62): 7112 ± 6454

Sample size estimation by statistical expertise (80% power, 5% risk level; two-sided test) suggests 141 patients in each group. To compensate for possible missed sampling and other causes for loss of data we plan to include a total of 310 patients.

Interim analysis will be performed by an independent external statistician after 160 patients in a way that does not increase the demands regarding statistical significance of the primary endpoints. An adaptive design implying that a surrogate variable (increase of NT-proBNP from preoperative level to postoperative day 1) known to correlate well with the primary endpoint will be used. Furthermore, regardless of if statistical significance is reached the study will proceed until 310 patients have been included or until the expiry date of the study solutions. Stopping criteria are given on page 12.

Continuous variables will analyzed with a two-tailed t-test or Mann-Whitney U test depending on the distribution of data.

Categorical variables will be analyzed with a two-tailed Chi square test unless expected cell counts are less than 5, then Fisher’s exact test will be used.

**Dropouts, missed sampling and exclusions**

Patients will be analyzed according to intention to treat.

Exclusions are estimated to be less than 1%. In the first Glutamics trial four patients out 865 who accepted participation were excluded. Dropouts and exclusions will be described when the study is published.

Missed sampling (NT-proBNP) on postoperative day 3 should lead to sampling the following day.

**Schedule and performance**

Funding applications to the Swedish Research Council and the Swedish Heart Lung Foundation have been submitted several times. Sufficient funding for the original version of the trial has not been achieved. The funding received has been approved for the downsized version of GLUTAMICS II by the Swedish Heart Lung Foundation. APL has started preparation for production of infusion solutions which are estimated to be available for delivery in October 2015. Because of restricted funding all study solutions (n=400) will be produced in a single batch with 24 months expiry date.

# Information to patients and written informed consent

# All patients eligible for the study will receive oral and written information about the trial. Written informed consent will be obtained in which the patient also is asked to permit potential independent audit in the future.

# Ethical considerations

Postoperative heart failure remains the main cause for prolonged intensive care, multiorgan failure and death after cardiac surgery(1). Available data suggest that glutamate can enhance the normal recovery of the heart after ischemia, which is associated with less strain on the myocardium than by stimulating myocardial contractility with conventional inotropic drugs. The potential benefits could therefore be substantial.

The patients will apart from an intravenous infusion of 300-500 mL of glutamate or normal saline receive normal postoperative care.

Glutamate is an amino acid naturally present in the human body and one of the most abundantly present free amino acids in the cardiomyocytes. During ischemia glutamate levels in the cardiomyocytes are depleted, which the heart compensates by extracting glutamate from the blood(6, 9). The extraction rate from plasma is approximately 50% postoperatively(14). The plasma levels increase rapidly during oral and intravenous administration(10, 11, 15, 16). In susceptible individuals an adverse reaction called Chinese restaurant syndrome consisting of transient flushing, headache and burning chest pain has occasionally been reported(17). In the first GLUTAMICS trial no adverse effects that could be directly linked to glutamate were observed(13).

In spite of the widespread use of glutamate there is no evidence that exogenous delivery of glutamate has caused neurological or other permanent complications in humans or primates(18-20). A possible cause is that glutamate does not pass the blood-brain barrier(19). Furthermore, the level of glutamate found in the central nervous system is up to 50 times higher than in blood(21). The glutamate infusion planned in the trial increases whole blood levels by 2-3-fold, which has been shown to increase myocardial uptake two-fold(22). Higher infusion rates have not been shown to further increase myocardial uptake of glutamate(22).

**Clinical importance and clinical experience**

Experience from the first GLUTAMICS trial suggests that glutamate could halve the risk of developing severe postoperative heart failure and thus cardiac mortality(13). According to O´Connor postoperative heart failure was the primary cause for mortality in 2/3 of the cases in Northern New England and the single most important cause for differences between institutions with good and bad results(1).

Our group has used glutamate in clinical practice at Linköping University Hospital for 20 years and our experience so far in CABG is associated with a mortality 75% lower than expected according to EuroSCORE. Compared with national and international results we noted that the risk of renal failure (probably the most sensitive indicator for the quality of postoperative heart failure treatment) after CABG has been at least five times lower (regardless of what criteria was used)(12, 23).

Patients admitted to surgery with severely depressed left ventricular function is the group of patients that has received prophylactic treatment most frequently. In a recently published study on 104 patients, we observed a mortality of 1.0% in a cohort with an expected mortality of 8.3% according to logistic EuroSCORE(12). As this type of results are influenced by other factors, they should be interpreted cautiously but they agree with the results of the first GLUTAMICS trial.

The biochemical properties of glutamate suggest that the clinical effects we aim to confirm in the GLUTAMICS II trial should be possible to emulate in studies on other patient cohorts with acute myocardial ischemia. The major clinical implications in the future may thus be found in acute myocardial infarction and other conditions characterized by acute myocardial ischemia(18, 24).

**Overall assessment of risk and benefit**

Glutamate has metabolic properties that can increase myocardial tolerance to ischemia and enhance recovery of metabolism and function after ischemia(2, 6). These properties are desirable when conventional pharmacologic al treatment and myocardial protection is insufficient. The need for alternative treatment is strengthened by the fact that traditional treatment of postoperative heart failure with inotropic drugs increase myocardial oxygen consumption excessively in relation to the effect on myocardial contractility and hence carries a risk of aggravating underlying ischemic myocardial injury(2, 25, 26). This could explain why postoperative heart failure has been identified as the single most important cause for postoperative mortality after CABG(1). In the first GLUTAMICS trial a relative risk reduction exceeding 50% of developing heart failure leading to extended intensive care or death was observed in most high-risk groups (table 1)(13).

Potential hazards include the adverse reactions reported on pages 11-12. The risk of adverse reactions, such as Chinese restaurant syndrome, asthma or urticaria is considered small with the dosages employed and will be further reduced by excluding patients with ambiguous food allergies. In our twenty-year experience with glutamate including the first GLUTAMICS trial we have so far not observed any of these adverse reactions. In light of this we consider the potential benefits to outweigh the potential hazards.

The investigators manual provides more details and references.

**Administration of glutamate**

Glutamate levels in plasma increase rapidly after both oral and intravenous administration(10, 15, 16, 22, 27). In association with cardiac surgery glutamate has been administered as an additive in cardioplegic solutions or intravenously. Experimental data suggest that transport of glutamate across the cellular membrane of cardiomyocytes is impeded by cardioplegia (hyperkalemic solution) and that the myocytes actually leak glutamate in the cardioplegic state(28). The main reason for choosing the intravenous route for administration of glutamate is our objective to enhance recovery during the first hours of reperfusion.

**Dosage of glutamate**

Available data demonstrate that myocardial uptake of glutamate correlates strongly with arterial levels in patients early after CABG(14). We also know that myocardial uptake of glutamate increases when plasma levels are increased by intravenous administration(10, 11). However, increasing arterial levels (whole blood) by more than 2-3-fold is not associated with a further increase of myocardial uptake. These arterial levels can be achieved with an infusion rate of 1.65 mL/ kg body weight and hour of a 0.125M glutamic acid solution(22).

The total volume to be infused according to plan (see research plan page 5) is estimated as follows depending on body weight: 70 kg - 289 mL; 80 kg - 330 ml; 90 kg - 371 mL; 100 kg - 412 mL. The maximum volume that can be infused is 500 mL.

Details about the composition of the solution is given in the Pharmaceutical documentation.

**Discontinuation of the infusion**

Infusion should be terminated if there are signs of allergic reaction (rash, bronchial obstruction, anaphylactic reaction) or SUSAR are encountered.

# Drug management

Drug management will follow the instructions from the producer APL

**Randomization, treatment of controls, patient identification**

Randomization is planned to be done in varying block sizes by APL in Umeå, the manufacturer of the study solutions.

The infusion bottle will have two copies of the randomization code, one will be on a self-adhesive label which should be removed and transferred to the CRF when the infusion is started. This is the point when the patient is included in the trial.

Masking will be double-blind. Placebo consists of normal saline. Both the glutamate solution and placebo are clear transparent solutions implying that you cannot tell one from the other visually. The study bottles are marked with a patient code that is registered in the CRF and in the institutional data base. A patient identification list will permit identification of patients in association with data check and quality control

The randomization codes are stored at APL in Umeå. Codes can be broken for individual patients in cases of suspect adverse reactions. This decision is made after consultation with the principal investigator or his deputy.

**Safety monitoring**

Postoperatively all patients are continually monitored with invasive hemodynamic monitoring (CVP, arterial blood pressure), electrocardiogram, diuresis, level of consciousness, discharge from chest tubes. Hemodynamically unstable patients are monitored with pulmonary artery catheters and SvO2 measurements. Transesophageal echocardiography is routinely performed and whenever patients are hemodynamically unstable. After the first day the level of monitoring is adjusted to the needs of the patient with regard to hemodynamic state. Early mobilization is desirable. Neurological state is controlled at awakening from anesthesia and a brain scan is performed if there are any signs of neurological injury. Biomarkers for myocardial injury (CK-MB) are checked on the first postoperative morning. Biomarkers for heart failure are checked on the first and third postoperative morning (NT-proBNP).

**Adverse reactions and toxicology**

Glutamate levels in plasma increase rapidly both after oral and intravenous administration. Despite widespread use of this substance, mainly as a flavor enhancer but also in infusion solutions there is currently no documentation showing that exogenously administered glutamate has caused permanent injury in man or primates(18-20).

Conventional toxicity studies on several species have yet to reveal any carcinogenic or teratogenic effects. No influence on reproduction has been found(29).

In the central nervous system glutamate is one of the dominating neurotransmitters. Under certain conditions glutamate may act as an excitotoxin and contribute to neuron damage and neuron death in association with acute and chronic neurological disease. Glutamate has been claimed to be involved in the pathogenesis associated with ischemic brain injuries and research is directed at finding glutamate receptor antagonists that might mitigate the injury(30).

Damage on the central nervous system has bee induced in certain species, mainly rodents, with large doses of exogenously administered glutamate(31). However, despite widespread use there is no evidence that exogenously administered glutamate has caused permanent neurological injury in humans or primates(18-20). A possible explanation is that glutamate does not pass the blood-brain barrier or placenta(19, 20).

Theoretically extracorporeal circulation used during cardiac surgery might influence the permeability of the blood brain barrier in a way that could influence the passage of glutamate.

However, at certain institutions glutamate has been used routinely as an additive in cardioplegic solutions (which increases plasma levels much more than what is achieved with the dosage used in our study) without any evidence for increased risk of complications from the central nervous system(32). A multivariable analysis of central nervous system complications did not reveal any tendency to increased risk of sustaining central nervous system complications after cardiac surgery related to glutamate. On the contrary a tendency to the opposite was observed(33).

In a neurotrauma cohort conventional amino acid solutions containing glutamate 3.75 gram/L was found to increase plasma levels of glutamate twice but no difference in outcome was found between patients receiving amino acids vs those not receiving amino acids(34).

In this context it might be worth considering that glutamate concentrations in the central nervous system are approximately 50 times higher than in blood(21). The glutamate infusion planned in our trial increases blood levels 2-3-fold(22).

In the first GLUTAMICS-trial involving 861 patients the risk for subclinical neurological injury was evaluated in a subgroup by sampling of biomarker S-100B. No differences or trends were found between the glutamate and control group regarding S-100B or clinical neurological events(35).

Despite being a natural amino acid in the human body adverse reactions and allergy has been discussed. It is mainly three conditions that has caught the eye in the medical literature. These are Chinese restaurant syndrome, glutamate elicited asthma and glutamate elicited urticaria. Chinese restaurant syndrome has received most attention (flushing, headache and chest pain). Several blinded studies, some of them done on patients who have claimed to have experienced symptoms related to glutamate, have failed to prove the connection as no difference in symptoms were found between patients receiving glutamate or not(36-38). Similar findings have been done in patients claimed to have glutamate elicited asthma(39, 40). There are studies suggesting that glutamate could cause Chinese restaurant syndrome in susceptible individuals(17). Although, there is reason to question the latter studies we have decided that ambiguous food allergies known to have elicited flushing, headache, rash or asthma will be exclusion criteria in the planned trial.

More details and references are given in the supplement about adverse reactions and toxicology.

**Adverse events (AE)**

Adverse events (AE) are common after cardiac surgery as all patients have some degree of postoperative pain, bleeding and fever. Serious adverse events (SAE) are defined as AE that have been life threatening or deadly or resulted in extended hospital care or permanent sequele. SAE are also common after cardiac surgery. Postoperative atrial fibrillation, the most common cause for exended hospital stay, is observed in approximately one third of the patients undergoing CABG.

In GLUTAMICS II AE will be registered and dived into AE and SAE. To determine if the study substance had any influence on AE these events will be compared between treatment group and control group. AE will be reported according to what organ has been affected: Central nervous system, heart - circulation, lungs, gastrointestinal tract, kidneys, skin. Infections will be reported according to would or organ affected. Postoperative nausea will be reported if it required antiemetic treatment.

**Reporting of Adverse events, side effects and SUSAR**

# ” Serious adverse events” (SAE) and SUSAR will be reported on a dedicated fax form to the Sponsor within 24 hours after the investigator gains knowledge about the event. Rules for breaking randomization codes are specified in the investigator’s manual.

Suspicion of side effects or SUSAR will be reported to the Swedish Medical Product Agency according to their instructions. The Sponsor is responsible for this task and will report SUSAR that have been life threatening or deadly withing 7 days and the remaining within 15 days.

Suspicion of side effects will also be reported to the local ethical review board.

Sponsor plans a yearly report of AE to the Swedish Medical Product Agency.

**Monitoring, quality control and interim analyses**

Participating centers will monitor each other 100% with regard to correct inclusion, written informed consent, primary and secondary endpoints, SAE and SUSAR. Complete monitoring regarding management of infusion solutions and original data is planned 10%.

The trial will be monitored internally on a weekly basis with regard to inclusions, completeness of sampling, management and storage of CRF and exclusions.

External statistical expertise at Linköping University will perform interim analysis, which is planned after 160 patients have been included in the trial.

The Swedish Medical Product Agency can perform an independent audit without prior notice.

**Clinical Endpoints committee**

The need for a clinical endpoints committe has been abolished with the novel downscaled design of GLUTAMICS II.

**Stopping criteria**

If monitoring or interim analysis would give suspicion of an increased risk for stroke or a negative impact on mortality the trial will be prematurely stopped. If monitoring indicates unexpected and serious adverse sideeffects the trial will be prematurely stopped. Safety aspect will thus have priority and the trial will be stopped before the incidence of serious side effects reraches statistical significance. In a situation when the next event would give a p-value < 0.10 with regard to a negative effect on stroke or mortality this issue will be discussed with the Swedish Medical Product Agency.

Sample size will be adjusted after interim analysis. If a positive effect on the surrogate marker for the primary endpoint has been achieved at interim analysis the study will still continue to a total of 310 patients or until the expiry date of the study solutions. The trial will be stopped if there is no absolute difference in the surrogate endpoint in favor of the glutamate group at interim analysis. Patients will be included in the trial while we await the outcome of the interim analysis.

**Insurances**

Heart surgery is associated with risks for complications and postoperative pain. It is, however, unlikely that participation in the trial will influence that negatively. The regular patient insurance is therefore considered sufficient.

**Information to staff**

Staff at all levels will be informed orally about the trial, its background and aims. Contact persons will be selected that will be available continuously for questions that arise. Furthermore, written information will be available at all units.

**Sponsor**

Rolf Svedjeholm, Thoraxkir sektionen, Thorax-kärlkliniken, US, Linköping

**Investigators**

Rolf Svedjeholm, överläkare, Thoraxkir sektionen, Thorax-kärlkliniken, US, Linköping

Jonas Holm, överläkare, Thoraxkir, Thorax-kärlkliniken, US, Linköping

Farkas Vanky, överläkare, Thoraxkir sektionen, Thorax-kärlkliniken, US, Linköping

Örjan Friberg, överläkare, Thoraxkir sektionen, Thoraxkliniken, US, Örebro

Gabriele Ferrari, ST-läkare, Thoraxkir sektionen, Thoraxkliniken, US, Örebro

Lars Algotsson, överläkare, Kliniken för thoraxkirurgi, anestesi och intensivvård, Skånes Universitetssjukhus, Lund.

Anders Holmgren, överläkare, Thoraxkirurgiska enheten, Norrlands Universitetssjukhus, Umeå

David Wariaro, överläkare, Thoraxanestesi, Norrlands Universitetssjukhus, Umeå

Andreas Borsiin, ST-läkare, Thoraxcentrum, Blekingesjukhuset, Karlskrona

Lena Jideus, överläkare, Verksamhetsområdet Thoraxkirurgi och Anestesi, Akademiska sjukhuset, Uppsala

**Responsibilities regarding delegation within the investigator group**

Responsible investigator delegates tasks to coworkers within the investigator group

**Reporting and publication**

The results will be made public regardless of outcome and will be submitted for peer review in an international cardiovascular/ cardiology/ heart surgery journal. Choice of journal will be discussed by the investigator group when the results are available.

Preliminary report of the results at an international conference is a possibility.

**Archiving of original data**

Primary data will be archived and will be available for independent externa audit for 15 years. Archiving will account for protection of patient secrecy and integrity. CRF:s will be archived in a locked space and will not permit identification of individual patients without access to the patient identification list which be kept locked in a separate space.

# Selected publications by the investigaors related to the trial

1. Svensson SE, Svedjeholm R, Ekroth R, Milocco I, Nilsson F, Sabel K G, William-Olsson G. Trauma metabolism and the heart; uptake of substrates and effects of insulin early after cardiac surgery. J Thorac Cardiovasc Surg 1990; 99: 1063 - 1073.

2. Svedjeholm R, Svensson SE, Ekroth R, Milocco I, Nilsson F, Vinnars E, Wernerman J. Trauma metabolism and the heart; studies of heart and leg amino acid flux after cardiac surgery. Thorac Cardiovasc Surgeon. 1990; 38: 1-5.

3. Svedjeholm R, Ekroth R, Joachimsson PO, Ronquist G, Svensson SE, Tydén H. Myocardial uptake of amino acids and other substrates in relation to myocardial oxygen consumption four hours after cardiac surgery. J Thorac Cardiovasc Surg 1991; 101: 688 - 694.

4. Svedjeholm R, Ekroth R, Joachimsson PO, Tydén H. High dose insulin improves the efficacy of dopamine early after cardiac surgery. A study of myocardial performance and oxygen consumption. Scand J Thorac Cardiovasc Surg. 1991; 25: 215 - 221.

5. Svedjeholm R, Hallhagen S, Ekroth R, Joachimsson PO, Ronquist G. Dopamine and High-dose Insulin infusion (Glucose-Insulin-Potassium) after a Cardiac Operation. Effects on Myocardial Metabolism. Ann Thorac Surg 1991; 51: 262 - 270.

6. Svedjeholm R, Huljebrant I, Håkanson E, Vanhanen I. The Rationale for Metabolic Support with Amino Acids and GIK (Glucose-Insulin-Potassium) in Cardiac Surgery. Ann Thorac Surg.1995; 59:S15-22.

7. Svedjeholm R, Huljebrant I, Håkanson E, Vanhanen I. Glutamate and high-dose glucose-insulin-potassium (GIK) in the treatment of severe cardiac failure after cardiac operations. Ann Thorac Surg. 1995; 59: S23-30.

8. Svedjeholm R, Vanhanen I, Håkanson E, Joachimsson PO, Jorfeldt L, Nilsson L. Metabolic and hemodynamic effects of intravenous glutamate infusion early after coronary surgery. J.Thorac.Cardiovasc.Surg. 112(6):1468-1477, 1996.

9. Vanhanen I, Svedjeholm R, Håkanson E, Joachimsson PO, Jorfeldt L, Nilsson L, Vanky F. Assessment of myocardial glutamate requirements early after coronary artery bypass surgery. Scand Cardiovasc J 1998; 32: 145-152.

10. Vanhanen I, Håkanson E, Jorfeldt L, Svedjeholm R. Myocardial uptake and release of substrates in patients operated for unstable angina: impact of glutamate infusion. Scand Cardiovasc J 2003; 37: 113-120.

11. Rolf Svedjeholm, Mårten Vidlund, Ingemar Vanhanen, Erik Håkanson. A metabolic protective strategy could improve long-term survival in patients with LV-dysfunction undergoing CABG. Scand Cardiovasc J 2010; 44: 45-58.

12. Mårten Vidlund, Jonas Holm, Erik Håkanson, Örjan Friberg, Lena Sunnermalm, Farkas Vanky, Rolf Svedjeholm. The S-100B substudy of the GLUTAMICS-trial: Glutamate infusion not associated with sustained elevation of plasma S-100B after coronary surgery. Clin Nutr 2010; 29: 358-64.

13. Mårten Vidlund, Erik Håkanson, Örjan Friberg, Sören Juhl-Andersen, Jonas Holm, Farkas Vanky, Lena Sunnermalm, Jan-Olov Borg, Rajiv Sharma, Rolf Svedjeholm. GLUTAMICS – a randomized clinical trial on glutamate infusion in patients operated for acute coronary syndrome. American College of Cardiology, New Orleans, April 2-5, 2011. (JACC Volume: 57   Issue: 14   Supplement: 1   Pages: E938-E938   Published: APR 5 2011)

14. Mårten Vidlund: Glutamate for metabolic intervention in coronary surgery – with special reference to the GLUTAMICS-trial. Thesis 2011: Örebro Studies of medicine 58.

**Table 1**

|  | **Number**  **Control/**  **Glutamate** | **Control** | **Glutamate** | **RR** | **95% CI** | **p-value** |
| --- | --- | --- | --- | --- | --- | --- |
| Isolated CABG | 413/411 | 16 (3.9%) | 6 (1.5%) | 0.38 | 0.15-0.95 | 0.04 |
| NSTEMI only | 68/59 | 1 (1.5%) | 1 (1.7%) | 1.15 | 0.07-18 | 0.92 |
| Diabetics | 108/98 | 4 (3.7%) | 4 (4.1%) | 1.10 | 0.28-4.3 | 0.89 |
| Non-diabetics | 305/313 | 12 (3.9%) | 2 (0.6%) | 0.16 | 0.04-0.72 | 0.02 |
| CSS class IV | 233/225 | 16 (6.9%) | 3 (1.3%) | 0.19 | 0.06-0.66 | 0.008 |
| Left main stenosis >50% | 141/174 | 10 (7.1%) | 1 (0.6%) | 0.08 | 0.01-0.63 | 0.02 |
| Female gender | 77/70 | 7 (8.5%) | 2 (2.7%) | 0.31 | 0.26-1.2 | 0.14 |
| EuroSCORE≥8 | 63/83 | 9 (14.3%) | 4 (4.8%) | 0.34 | 0.11-1.05 | 0.06 |
| Severe LV-dysfunction | 20/14 | 5 (25%) | 1 (7.1%) | 0.29 | 0.04-2.2 | 0.23 |
| Emergency* | 13/14 | 4 (31%) | 1 (7.1%) | 0.23 | 0.03-1.6 | 0.16 |
| LV-failure  at weaning | 14/18 | 8 (57%) | 2 (11%) | 0.19 | 0.05-0.78 | 0.02 |

**Table 1.**  The incidence of severe postoperative circulatory failure in the control group and glutamate group in different subgroups undergoing isolated CABG. The relative risk ± 95% confidence interval of developing severe postoperative circulatory failure associated with glutamate treatment. Results are approximative given the small number of events. * Emergency according to EuroSCORE definition. RR= relative risk; CI= confidence interval.

**Table2.** Outcome data for patients with LV-failure at weaning from CPB (median+ interquartile range, mean±SD or count n %).

|  | *Placebo*  *n=18* | *Glutamate*  *n=20* | *p-value* |
| --- | --- | --- | --- |
| a-Lactate mmol/L 5 minutes after protamine | 2.5±1.0 | 1.7±0.5 | *0.004* |
| Hemodynamic state at completion of surgery |  |  |  |
| -Unstable with inotropes / IABP | 31% (5/16) | 0 | *0.01* |
|  |  |  |  |
| Severe circulatory failure | 56%(10/18) | 15%(3/20) | *0.02* |
| Postop increase of p-Creatinine (Δ µmol/L) | 58±87 | 19±34 | 0.08 |
| Renal injury (increased creatinine x2 ) | 22% | 0% | *0.03* |
| Ventilator treatment (hours) | 7.4 [5.8-49] | 5.0 [3.3-8.0] | *0.02* |
| ICU stay (hours) | 92 [41-139] | 25 [18-57] | *0.02* |
|  |  |  |  |

**Figure 1. Postoperative NT-proBNP (day 1) v severe heart failure.**


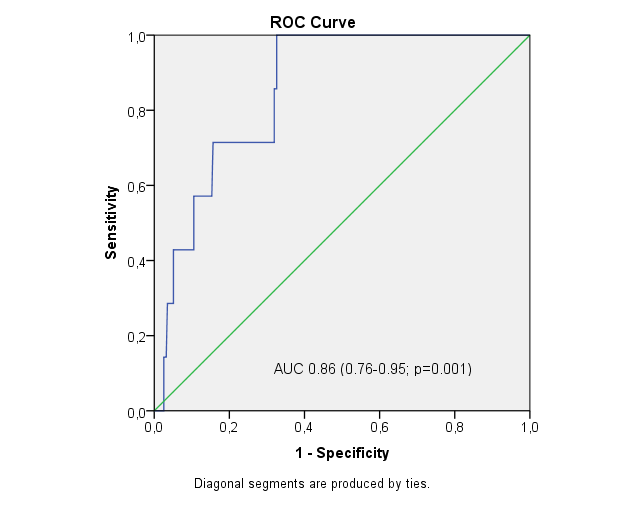


**References**

1. O'Connor GT, Birkmeyer JD, Dacey LJ, Quinton HB, Marrin CA, Birkmeyer NJ, et al. Results of a regional study of modes of death associated with coronary artery bypass grafting. Northern New England Cardiovascular Disease Study Group. Ann Thorac Surg. 1998;66(4):1323-8.

2. Svedjeholm R, Hakanson E, Vanhanen I. Rationale for metabolic support with amino acids and glucose-insulin-potassium (GIK) in cardiac surgery. Ann Thorac Surg. 1995;59(2 Suppl):S15-22.

3. Mudge GH, Jr., Mills RM, Jr., Taegtmeyer H, Gorlin R, Lesch M. Alterations of myocardial amino acid metabolism in chronic ischemic heart disease. J Clin Invest. 1976;58(5):1185-92.

4. Thomassen AR, Nielsen TT, Bagger JP, Henningsen P. Myocardial exchanges of glutamate, alanine and citrate in controls and patients with coronary artery disease. Clin Sci (Lond). 1983;64(1):33-40.

5. Rau EE, Shine KI, Gervais A, Douglas AM, E. C. Amos r. Enhanced mechanical recovery of anoxic and ischemic myocardium by amino acid perfusion. American Journal of Physiology-Heart and Circulatory Physiology. 1979;236(6):H873-H9.

6. Pisarenko OI. Mechanisms of myocardial protection by amino acids: facts and hypotheses. Clin Exp Pharmacol Physiol. 1996;23(8):627-33.

7. Lazar HL, Buckberg GD, Manganaro AJ, Becker H, Maloney JV, Jr. Reversal of ischemic damage with amino acid substrate enhancement during reperfusion. Surgery. 1980;88(5):702-9.

8. Burns AH, Reddy WJ. Amino acid stimulation of oxygen and substrate utilization by cardiac myocytes. American Journal of Physiology-Endocrinology and Metabolism. 1978;235(5):E461.

9. Pisarenko OI, Oleynikov OD, Shulzhenko VS, Studneva IM, Ryff IM, Kapelko VI. Association of myocardial glutamate and aspartate pool and functional recovery of postischemic heart. Biochem Med Metab Biol. 1989;42(2):105-17.

10. Pisarenko OI, Lepilin MG, Ivanov VE. Cardiac metabolism and performance during L-glutamic acid infusion in postoperative cardiac failure. Clin Sci (Lond). 1986;70(1):7-12.

11. Svedjeholm R, Vanhanen I, Hakanson E, Joachimsson PO, Jorfeldt L, Nilsson L. Metabolic and hemodynamic effects of intravenous glutamate infusion early after coronary operations. J Thorac Cardiovasc Surg. 1996;112(6):1468-77.

12. Svedjeholm R, Vidlund M, Vanhanen I, Hakanson E. A metabolic protective strategy could improve long-term survival in patients with LV-dysfunction undergoing CABG. Scand Cardiovasc J. 2010;44(1):45-58.

13. Vidlund M, Hakanson E, Friberg O, Juhl-Andersen S, Holm J, Vanky F, et al. GLUTAMICS--a randomized clinical trial on glutamate infusion in 861 patients undergoing surgery for acute coronary syndrome. J Thorac Cardiovasc Surg. 2012;144(4):922-30 e7.

14. Svedjeholm R, Ekroth R, Joachimsson PO, Ronquist G, Svensson S, Tydén H. Myocardial uptake of amino acids and other substrates in relation to myocardial oxygen consumption four hours after cardiac operations. J Thorac Cardiovasc Surg. 1991;101(4):688-94.

15. Fernstrom JD. Pituitary hormone secretion in normal male humans: acute responses to a large, oral dose of monosodium glutamate. J Nutr. 2000;130(4S Suppl):1053s-7s.

16. Thomassen A, Nielsen TT, Bagger JP, Pedersen AK, Henningsen P. Antiischemic and metabolic effects of glutamate during pacing in patients with stable angina pectoris secondary to either coronary artery disease or syndrome X. Am J Cardiol. 1991;68(4):291-5.

17. Geha RS, Beiser A, Ren C, Patterson R, Greenberger PA, Grammer LC, et al. Review of alleged reaction to monosodium glutamate and outcome of a multicenter double-blind placebo-controlled study. J Nutr. 2000;130(4S Suppl):1058s-62s.

18. Thomassen AR. Myocardial uptake and effects of glutamate during non-ischaemic and ischaemic conditions. A clinical study with special reference to possible interrelationships between glutamate and myocardial utilization of carbohydrate substrates. Dan Med Bull. 1992;39(6):471-88.

19. Sacks W, Sacks S, Brebbia DR, Fleischer A. Cerebral uptake of amino acids in human subjects and rhesus monkeys in vivo. J Neurosci Res. 1982;7(4):431-6.

20. Stegink LD, Pitkin RM, Reynolds WA, Filer LJ, Jr., Boaz DP, Brummel MC. Placental transfer of glutamate and its metabolites in the primate. Am J Obstet Gynecol. 1975;122(1):70-8.

21. Waelsch H. Glutamic acid and cerebral function. Adv Protein Chem. 1951;6:299-341.

22. Vanhanen I, Svedjeholm R, Håkanson E, Joachimsson PO, Jorfeldt L, Nilsson L, et al. Assessment of myocardial glutamate requirements early after coronary artery bypass surgery. Scand Cardiovasc J. 1998;32(3):145-52.

23. Svedjeholm R, Hakanson E, Szabo Z. Routine SvO2 measurement after CABG surgery with a surgically introduced pulmonary artery catheter. Eur J Cardiothorac Surg. 1999;16(4):450-7.

24. Thomassen AR, Mortensen PT, Nielsen TT, Falstie-Jensen N, Thygesen K, Henningsen P. Altered plasma concentrations of glutamate, alanine and citrate in the early phase of acute myocardial infarction in man. Eur Heart J. 1986;7(9):773-8.

25. Fellahi JL, Parienti JJ, Hanouz JL, Plaud B, Riou B, Ouattara A. Perioperative use of dobutamine in cardiac surgery and adverse cardiac outcome: propensity-adjusted analyses. Anesthesiology. 2008;108(6):979-87.

26. Svedjeholm R, Ekroth R, Joachimsson PO, Tydén H. High-dose insulin improves the efficacy of dopamine early after cardiac surgery. A study of myocardial performance and oxygen consumption. Scand J Thorac Cardiovasc Surg. 1991;25(3):215-21.

27. Fernstrom JD. Monosodium Glutamate in the Diet Does Not Raise Brain Glutamate Concentrations or Disrupt Brain Functions. Ann Nutr Metab. 2018;73 Suppl 5:43-52.

28. Kimose HH, Ravkilde J, Helligsö P, Knudsen MA, Thomassen AR, Nielsen TT, et al. Myocardial loss of glutamate after cold chemical cardioplegia and storage in isolated blood-perfused pig hearts. Thorac Cardiovasc Surg. 1993;41(2):93-100.

29. Walker R, Lupien JR. The safety evaluation of monosodium glutamate. J Nutr. 2000;130(4S Suppl):1049s-52s.

30. Greenamyre JT, Porter RH. Anatomy and physiology of glutamate in the CNS. Neurology. 1994;44(11 Suppl 8):S7-13.

31. Daabees TT, Finkelstein MW, Stegink LD, Applebaum AE. Correlation of glutamate plus aspartate dose, plasma amino acid concentration and neuronal necrosis in infant mice. Food Chem Toxicol. 1985;23(10):887-93.

32. Loop FD, Higgins TL, Panda R, Pearce G, Estafanous FG. Myocardial protection during cardiac operations. Decreased morbidity and lower cost with blood cardioplegia and coronary sinus perfusion. J Thorac Cardiovasc Surg. 1992;104(3):608-18.

33. Svedjeholm R, Håkanson E, Szabó Z, Vánky F. Neurological injury after surgery for ischemic heart disease: risk factors, outcome and role of metabolic interventions. Eur J Cardiothorac Surg. 2001;19(5):611-8.

34. Stover JF, Kempski OS. Glutamate-containing parenteral nutrition doubles plasma glutamate: a risk factor in neurosurgical patients with blood-brain barrier damage? Crit Care Med. 1999;27(10):2252-6.

35. Vidlund M, Holm J, Håkanson E, Friberg O, Sunnermalm L, Vanky F, et al. The S-100B substudy of the GLUTAMICS trial: glutamate infusion not associated with sustained elevation of plasma S-100B after coronary surgery. Clin Nutr. 2010;29(3):358-64.

36. Wilkin JK. Does monosodium glutamate cause flushing (or merely "glutamania")? J Am Acad Dermatol. 1986;15(2 Pt 1):225-30.

37. Prawirohardjono W, Dwiprahasto I, Astuti I, Hadiwandowo S, Kristin E, Muhammad M, et al. The administration to Indonesians of monosodium L-glutamate in Indonesian foods: an assessment of adverse reactions in a randomized double-blind, crossover, placebo-controlled study. J Nutr. 2000;130(4S Suppl):1074s-6s.

38. Tarasoff L, Kelly MF. Monosodium L-glutamate: a double-blind study and review. Food Chem Toxicol. 1993;31(12):1019-35.

39. Woessner KM, Simon RA, Stevenson DD. Monosodium glutamate sensitivity in asthma. J Allergy Clin Immunol. 1999;104(2 Pt 1):305-10.

40. Woods RK, Weiner JM, Thien F, Abramson M, Walters EH. The effects of monosodium glutamate in adults with asthma who perceive themselves to be monosodium glutamate-intolerant. J Allergy Clin Immunol. 1998;101(6 Pt 1):762-71.
