# Supplementary material for: Effect of glutamate infusion on NT-proBNP after coronary artery bypass grafting in high-risk patients (GLUTAMICS II): A randomized controlled trial
Source: PLoS Med. 2022 May 9;19(5):e1003997. doi: 10.1371/journal.pmed.1003997 (PMC9126383; doi:10.1371/journal.pmed.1003997)
Supplement: S1 Supporting information — (DOCX) [file pmed.1003997.s003.docx]

S1 SUPPORTING INFORMATION

Effect of glutamate infusion on NT-proBNP after coronary artery bypass grafting in high-risk patients (GLUTAMICS II): A randomized controlled trial

Jonas Holm,^1^ Gabriele Ferrari,^2^ Anders Holmgren,^3^ Farkas Vanky,^1^ Örjan Friberg,^2^ Mårten Vidlund,^2,4^ Rolf Svedjeholm,^1^

**Clinical trial registration:** <https://clinicaltrials.gov/ct2/show/NCT02592824>

**Fig A. CONSORT DIAGRAM**

4959 patients underwent CABG with or without additional procedure and 430 patients were identified as potentially eligible between November 15, 2015 and September 30, 2020

321 patients gave written informed consent

314 randomized

Allocated to Glutamate infusion n=155

Allocated to Saline infusion n=159

Follow-up n=148

Lost CRF n=2

Analyzed n=148

Excluded from analysis n=0

Analyzed n=155

Excluded from analysis n=0

# **ALLOCATION**

FOLLOW-UP

ANALYSIS

ENROLLMENT

Not randomized

2 change of procedure

5 forgetfulness

Excluded due to intraoperative exclusion criteria n=5

Excluded due to intraoperative exclusion criteria n=3

Follow-up n=155

Lost CRF n=1

Received allocated intervention n=150

Received allocated intervention n=156

57 declined participation

39 exclusion criteria

5 surgery cancelled

4 other procedure (PCI/off pump)

2 other trial

1 declined surgery

1 emergency

**Fig A.** CONSORT diagram of patients included in the GLUTAMICS II trial.

Causes for patients not being analyzed are given in the figure.

Preoperative exclusion criteria: renal failure n=18, informed consent not possible because of critical condition or other reason n=15, cardiac risk factor did not fulfil study criteria n=3, age >85 years n=2, complex case n=1.

Intraoperative exclusion criteria in the glutamate group: change of procedure n=4 (off pump n=2, surgery on the ascending aorta n=1, planned valve procedure not performed n=1) incorrect inclusion n=1 (preoperative creatinine clearance < 30 mL/min).

Intraoperative exclusion criteria in the control group were: change of procedure n=1 (surgery of ascending aorta) Incorrect inclusion n=2 (age >85 years n=1, creatinine clearance <30 mL/min n=2). Details of intraoperatively excluded patients are given in the table below.

Lost CRF n=3 (case record forms missing). Screening and recruitment of patients was done by the investigators at participating sites. Screening in Gothenburg only involved patients operated by the participating surgeon. Correct inclusion was confirmed by the external monitoring team and loss to follow-up documented before the database was locked and submitted to an external statistician.

**Details of intraoperatively excluded patients**

| **Group** | **Exclusion criteria** | **ICU stay**  **days** | **NT-proBNP**  **preop** | **NT-proBNP**  **Day 3** | **Alive 1 year after surgery** |
| --- | --- | --- | --- | --- | --- |
| Control | Surgery of ascending aorta | 3 | 240 | 11300 | yes |
| Control | eCrCl < 30 mL•min^-1^ | 4 | 5370 | 28100 | yes |
| Control | Age > 85 years | 2 | 11760 | 22614 | yes |
| Glutamate | eCrCl < 30 mL•min^-1^ | 1 | 6921 | 17168 | yes |
| Glutamate | OPCAB | 1 | 2000 | 5820 | yes |
| Glutamate | Surgery of ascending aorta | 1 | 1570 | 3170 | yes |
| Glutamate | Change of procedure  (no valve) | 1 | 580 | - | yes |
| Glutamate | OPCAB | 1 | - | - | yes |

eCrCl: estimated Creatinine clearance according to Cockcroft-Gault formula; ICU = Intensive Care Unit; OPCAB = off pump coronary artery bypass

**Fig B.**


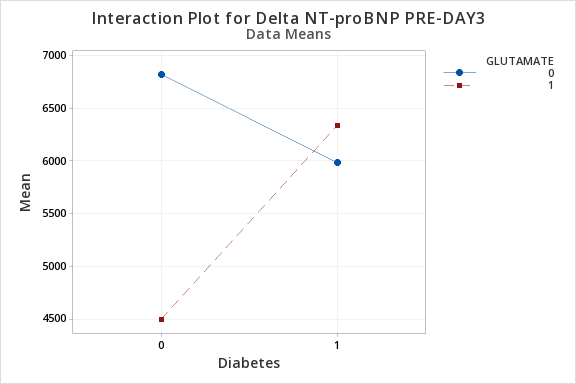


**Analysis of Variance**

| **Source** | **DF** | **Adj SS** | **Adj MS** | **F-Value** | ***P*-Value** |
| --- | --- | --- | --- | --- | --- |
| Glutamate | 1 | 71326910 | 71326910 | 2.56 | 0.110 |
| Diabetes | 1 | 18364377 | 18364377 | 0.66 | 0.417 |
| Glutamate*Diabetes | 1 | 130535705 | 130535705 | 4.69 | 0.031 |
| Error | 291 | 8097598159 | 27826798 |  |  |
| Total | 294 | 8328638223 |  |  |  |

**Fig B.** Interaction between Glutamate and Diabetes for postoperative rise of NT-proBNP preop to Day 3 according to Analysis of Variance (p=0.03)

**SUPPLEMENTARY TABLES**

**Table A.** Multivariable linear regression results for the association between glutamate and the difference increase of NT-proBNP from preoperative level to POD3 in patients without diabetes adjusted for age, eCrCl and EuroSCORE II

|  | B | 95% CI | *P* value | VIF |  |
| --- | --- | --- | --- | --- | --- |
| EuroSCORE II | 483 | 74 - 893 | 0.021 | 1.15 |  |
| Glutamate | - 1804 | -3427 - -182 | 0.029 | 1.03 |  |
| eCrCl (mL•min^-1^) | - 33 | -79 - 12 | 0.148 | 1.54 |  |
| Age (years) | 65 | -79 - 210 | 0.374 | 1.40 |  |

R^2^ = 0.14, ANOVA for the model (df = 4, F = 6.1, p<0.001) CI: confidence interval; eCrCl: preoperative estimated Creatinine clearance according to Cockcroft-Gault formula; VIF = variance inflation factor.

**Table B.** Multivariable analysis of the association between glutamate and the risk for acute kidney injury in patients without diabetes adjusted for age, eCrCl and EuroSCORE II

| Variable | Odds ratio | 95% CI | *P* value |
| --- | --- | --- | --- |
| Glutamate | 0.30 | 0.12 – .74 | 0.008 |
| Age (years) | 1.06 | 0.98 – 1.15 | 0.17 |
| EuroSCORE II | 0.92 | 0.73 – 1.16 | 0.47 |
| eCrCl (mL•min^-1^) | 1.004 | 0.98 – 1.03 | 0.78 |

Multivariable logistic regression model. Nagelkerke R^2^ = 0.11; Hosmer-Lemeshow goodness-of-fit test x^2^ (df=8)=7.68, *p*=.47. CI: confidence interval; eCrCl: estimated Creatinine clearance according to Cockcroft-Gault formula

**Table C.** Multivariable linear regression results for variables significantly associated with difference in NT-proBNP from preoperative level to POD3 in patients without diabetes

|  | B | 95% CI | *P* value | VIF |  |
| --- | --- | --- | --- | --- | --- |
| eCrCl (mL•min^-1^) | - 53 | -90 - -16 | 0.005 | 1.03 |  |
| CABG+valve | 2425 | 642 - 4209 | 0.008 | 1.03 |  |
| Glutamate | - 2062 | -3659 - -465 | 0.012 | 1.02 |  |
| NT-proBNP preop | - 0.31 | -0.59 - -0.25 | 0.033 | 1.01 |  |

R^2^ = 0.16, ANOVA for the model (df = 4, F = 7.4, p<0.001) CI: confidence interval; eCrCl: preoperative estimated Creatinine clearance according to Cockcroft-Gault formula; VIF = variance inflation factor.

**Table D.** Test of Between-Subjects Effects with log difference NT-proBNP from preoperative level to POD3 as dependent variable in patients without diabetes

|  | Type III  Sum of Squares | df | Mean Square | F | *P* value |  |
| --- | --- | --- | --- | --- | --- | --- |
| Corrected Model | 42.98 | 6 | 7.16 | 4.74 | <0.001 |  |
| Intercept | 78.49 | 1 | 78.49 | 51.94 | <0.001 |  |
| Glutamate | 10.59 | 1 | 10.59 | 7.01 | 0.009 |  |
| CABG+valve | 2.79 | 1 | 2.79 | 1.85 | 0.176 |  |
| Site | 12.40 | 3 | 4.13 | 2.74 | 0.046 |  |
| logNT-proBNP preop | 8.31 | 1 | 8.31 | 5.50 | 0.02 |  |
| Error | 228.2 | 151 |  |  |  |  |
| Total | 2130.7 | 158 |  |  |  |  |
| Corrected total | 271.2 | 157 |  |  |  |  |

R^2^ = 0.158 (Adjusted R Squared = 0.125

**Table E.** Preoperative characteristics of patients with diabetes in the glutamate group and in the control group (saline).

| Variables | Glutamate  (n=72) | Control  (n=71) |
| --- | --- | --- |
| Age, y | 72 ± 7 | 75 ± 7 |
| Female sex, No. (%) | 17 (24) | 20 (28) |
| BMI, kg•m^2^ | 29 ± 6 | 28 ± 4 |
| EuroSCORE II, mean (SD), % | 4.9 ± 2.4 | 5.7 ± 2.5 |
| Diabetes, No. (%) | 72 (100) | 71 (100) |
| Hypertension, No. (%) | 62 (86) | 60 (84) |
| COPD, No. (%) | 10 (14) | 9 (13) |
| Peripheral arterial disease, No. (%) | 13 (18) | 16 (22) |
| Cerebrovascular disease, No. (%) | 5 (7) | 7 (10) |
| p-Creatinine, µmol• L^-1^ | 102 ± 28 | 107 ± 33 |
| eCrCl, mL• min^-1^ | 70 ± 25 | 64 ± 25 |
| NT-proBNP, ng •L^-1^ | 2970 ± 5492 | 2697 ± 4120 |
| Left main stenosis, No. (%) | 23 (32) | 31 (44) |
| AMI≤3 weeks, No. (%) | 40 (55) | 38 (53) |
| CCS IV, No. (%) | 10 (14) | 15 (21) |
| Atrial fibrillation, No. (%) | 10 (14) | 10 (14) |
| Severe LV dysfunction, No. (%) | 19 (26) | 16 (22) |

AMI ≤ 3 weeks: acute myocardial infarction within 3 weeks of surgery; BMI: body mass index; CCS: Canadian cardiovascular society; COPD: chronic obstructive pulmonary disease; EuroSCORE II: European system for cardiac operative risk evaluation II; LV: left ventricular; eCrCl: estimated Creatinine clearance according to Cockcroft-Gault formula; STEMI: ST-elevation myocardial infarction.

**Table F.** Intraoperative and postoperative characteristics of patients with diabetes in the glutamate group and in the control group (saline).

| Variables | Glutamate  (n= 72) | Control  (n= 71) | *P value* |
| --- | --- | --- | --- |
| Urgent / emergent procedure, No. (%) | 57 (79) | 52 (73) | 0.44 |
| Number of bypasses | 3.6 ± 1.0 | 3.4 ± 1.0 | 0.46 |
| Additional valve procedure, No. (%) | 9 (12) | 12 (17) | 0.49 |
| Aortic crossclamp time, min | 70 ± 31 | 67 ± 30 | 0.56 |
| CPB time, min | 107 ± 40 | 107 ± 39 | 0.97 |
| NT-proBNP POD1, ng•L^-1^ | 4907 ± 5926 | 4518 ± 5083  n=69 | 0.68 |
| NT-proBNP POD3, ng•L^-1^ | 9310 ± 9803  n=70 | 8672 ± 6653  n=67 | 0.66 |
| NT-proBNP POD3-Pre, ng•L^-1^ | 6339 ± 5815  n=70 | 5991 ± 4587  n=67 | 0.70 |
| CK-MB POD1, µg•L^-1^ | 17 [12-27] | 18 [12-24] | 0.86 |
| ICU stay, days | 1 [1-2] | 1 [1-2] | 0.97 |
| Ventilation time, h | 4.7 [2.7-7.3] | 4.5 [3.1-7.1] | 0.97 |
| Ventilation time >48 h, No. (%) | 6 (8.3) | 2 (2.9) | 0.27 |
| IABP, No. (%) | 1 (1.4) | 1 (1.4) | 1 |
| Reoperation bleeding, No. (%) | 6 (8.3) | 8 (11) | 0.59 |
| Postoperative AFib, No. (%) | 27 (37) | 32 (45) | 0.40 |
| Postop stroke ≤ 24hrs, No. (%) | 0 (0) | 2 (2.8) | 0.24 |
| AKI, No. (%) | 18 (25) | 17 (24) | 0.85 |
| Mortality ≤ 30 days, No. (%) | 1 (1.4) | 5 (7.0) | 0.12 |

AFib: atrial fibrillation; AKI: acute kidney injury; CK-MB: creatine kinase-MB isoenzyme; CPB: cardiopulmonary bypass; IABP: intra-aortic balloon pump; ICU: intensive care unit; POD: postoperative day

**Table G.** Preoperative characteristics of patients undergoing CABG + additional valve procedure in the glutamate group and in the control group (saline).

| Variables | Glutamate  (n=32) | Control  (n=33) |
| --- | --- | --- |
| Age, y | 76 ± 5 | 76 ± 5 |
| Female sex, No. (%) | 8 (25) | 9 (27) |
| BMI, kg•m^2^ | 26 ± 5 | 28 ± 5 |
| EuroSCORE II, mean (SD), % | 5.1 ± 1.9 | 6.3 ± 3.3 |
| Diabetes, No. (%) | 9 (28) | 12 (36) |
| Hypertension, No. (%) | 25 (78) | 25 (76) |
| COPD, No. (%) | 8 (25) | 4 (12) |
| Peripheral arterial disease, No. (%) | 4 (12) | 6 (18) |
| Cerebrovascular disease, No. (%) | 1 (3) | 3 (9) |
| p-Creatinine, µmol• L^-1^ | 109 ± 28 | 98 ± 26 |
| eCrCl, mL• min^-1^ | 60 ± 14 | 64 ± 19 |
| NT-proBNP, ng •L^-1^ | 2520 ± 4767 | 2538 ± 2371 |
| Left main stenosis, No. (%) | 11 (34) | 9 (27) |
| AMI≤3 weeks, No. (%) | 7 (22) | 12 (36) |
| CCS IV, No. (%) | 1 (3) | 1 (3) |
| Atrial fibrillation, No. (%) | 6 (19) | 7 (21) |
| Severe LV dysfunction, No. (%) | 1 (3) | 4 (12) |

AMI ≤ 3 weeks: acute myocardial infarction within 3 weeks of surgery; BMI: body mass index; CCS: Canadian cardiovascular society; COPD: chronic obstructive pulmonary disease; EuroSCORE II: European system for cardiac operative risk evaluation II; LV: left ventricular; eCrCl: estimated Creatinine clearance according to Cockcroft-Gault formula; STEMI: ST-elevation myocardial infarction.

**Table H.** Intraoperative and postoperative characteristics of patients undergoing CABG + additional valve procedure in the glutamate group and in the control group (saline).

| Variables | Glutamate  (n= 32) | Control  (n= 33) | *P value* |
| --- | --- | --- | --- |
| Urgent / emergent procedure, No. (%) | 12 (37) | 17 (52) | 0.32 |
| Number of bypasses | 3.2 ± 1.3 | 3.1 ± 0.8 | 0.80 |
| Additional valve procedure, No. (%) | 32 (100) | 33 (100) | 1 |
| Aortic crossclamp time, min | 113 ± 25 | 117 ± 31 | 0.58 |
| CPB time, min | 159 ± 37 | 162 ± 37 | 0.75 |
| NT-proBNP POD1, ng•L^-1^ | 5255 ± 3548 | 5795 ± 3380 | 0.53 |
| NT-proBNP POD3, ng•L^-1^ | 8616 ± 4774 | 10372 ± 7707 | 0.28 |
| NT-proBNP POD3-Pre, ng•L^-1^ | 6096 ± 6342 | 7764 ± 7205 | 0.33 |
| CK-MB POD1, µg•L^-1^ | 34 [18-65] | 31 [23-61] | 0.97 |
| ICU stay, days | 1 [1-3] | 2 [1-3] | 0.48 |
| Ventilation time, h | 3.8 [2.7-6.9] | 6.0 [3.0-18.3] | 0.32 |
| Ventilation time >48 h, No. (%) | 3 (9) | 3 (9) | 1 |
| IABP, No. (%) | 0 (0) | 1 (3) | 1 |
| Reoperation bleeding, No. (%) | 4 (12) | 8 (24) | 0.34 |
| Postoperative AFib, No. (%) | 15 (47) | 17 (52) | 0.81 |
| Postop stroke ≤ 24hrs, No. (%) | 0 (0) | 1 (3) | 1 |
| AKI, No. (%) | 6 (19) | 17 (52) | 0.009 |
| Mortality ≤ 30 days, No. (%) | 0 (0) | 1 (3) | 1 |

AFib: atrial fibrillation; AKI: acute kidney injury; CK-MB: creatine kinase-MB isoenzyme; CPB: cardiopulmonary bypass; IABP: intra-aortic balloon pump; ICU: intensive care unit; POD: postoperative day

**SUPPLEMENTARY METHODS**

**Statistical Analysis Plan**

The sample size is based on available results on NT-proBNP from the first GLUTAMICS-trial. In that study the following increase of NT-proBNP was observed in patients undergoing CABG or CABG with concomitant procedure who fulfilled inclusion criteria for the planned trial from preoperative values to postoperative day 3 (mean ± Standard Deviation)

Glutamate (n=71): 5261 ± 4409

Placebo (n=62): 7112 ± 6454

Sample size estimation by statistical expertise (80% power, 5% risk level; independent two-sided t-test assuming unequal variances) suggests 141 patients in each group. To compensate for possible missed sampling and other causes for loss of data we plan to include a total of 310 patients.

Interim analysis will be performed by an independent external statistician after 160 patients in a way that does not increase the demands regarding statistical significance of the primary endpoints. An adaptive design implying that a surrogate variable (increase of NT-proBNP from preoperative level to postoperative day 1) known to correlate well with the primary endpoint will be used. Furthermore, regardless of if statistical significance is reached the study will proceed until 310 patients have been included or until the expiry date of the study solutions. Stopping criteria are given in the Study protocol.

Continuous variables will analyzed with a two-tailed t-test or Mann-Whitney U test depending on the distribution of data.

Categorical variables will be analyzed with a two-tailed Chi square test unless expected cell counts are less than 5, then Fisher’s exact test will be used.

Adjustment for extra-cardiac variables (age, gender, preoperative renal function, body mass index) known to influence NT-proBNP and preoperative cardiac function has been considered. However, we have decided to rely on this study as a pure randomised clincial trial without further statistical adjustment as it is based on a post-hoc analysis in a similar cohort and adequately powered.

**CLINICAL MANAGEMENT**

Patients were managed according to the regular clinical routine. Clinical management was standardised and similar at the four participating centres with minor differences concerning choice of anaesthetic drugs.

After an overnight fast patients received beta-blockers and calcium antagonists orally whereas antihypertensive and antidiabetic agents were withheld. Standard premedication consisted of paracetamol 1g and oxazepam 5-10 mg orally about one hour before arrival to the operating theatre.

Anaesthesia was induced with thiopentone (2-3 mg kg^-1^ bodyweight, BW) or propofol (2 mg kg-^1^ BW) supplemented by a bolus dose of fentanyl 3-5 µg kg^-1^ BW. Muscle relaxation was achieved with pancuronium 0.1 mg kg^-1^ BW or rocuronium 0.6 mg kg^-1^ BW.

Anaesthesia was maintained with isoflurane, sevoflurane or propofol supplemented with intermittent doses of fentanyl. Standard monitoring was used consisting of 5-lead echocardiogram, pulse oximetry, continuous arterial blood pressure monitoring using a cannula in the radial artery, central venous pressure and transoesophageal echocardiography. A urinary catheter (Foley No. 12) having a transducer was used to measure temperature

Standard surgical techniques were employed. A median sternotomy was performed in all patients. Surgery was done on the arrested heart employing cardiopulmonary bypass and aortic cross-clamping. Cold blood cardioplegia was used for myocardial protection.

Postoperative sedation was achieved with propofol. Routine postoperative analgesia consisted of ketobemidone 7-15 µg kg^-1^ BW administered intermittently intravenously and acetaminophen 1 g every 6th hour.

The patients were extubated when body temperature had reached a level above 37°C, haemodynamic values were stable, PO_2_ was above 10 kPa with FiO_2_ 0.4, PCO_2_ was below 6.5 kPa with a respiratory rate less than 30, and drainage loss less than 100 mL per hour and declining.

Intra-operative and postoperative glycemic control was employed at all participating centers with a p-glucose target of 5-10 mmol/L (Örebro) or 6-10 mmol/L. Insulin infusion was started at p-glucose 8 mmol/L (Örebro) or 10 mmol/L (other centers).

After discharge from the ICU patients were transferred to a step-down semi-intensive care unit for at least 24 hours before going to the general ward.
